# Development and validation of a prediction model for invasive syndrome in liver abscess patients based on LASSO regression: a multi-center retrospective cohort study in China

**DOI:** 10.3389/fmed.2025.1572054

**Published:** 2025-11-28

**Authors:** Dian-Dian Hao, Guang-Zhao Shao, Xiu-Li Wang, Chang-Cheng Zhao, Jia-Lin Du, Hui Wang, Yu-Lin Ren, Yu-Ze Song, Xiao-Yu Wen

**Affiliations:** 1 Department of Hepatology, Center for Infectious Diseases and Pathogenic Biology, The First Hospital of Jilin University, Changchun, China; 2 Department of Hepatobiliary and Pancreatic Surgery, General Surgery Center, The First Hospital of Jilin University, Changchun, China; 3 Department of General Surgery, Yanshi People’s Hospital, Luoyang, Henan, China; 4 Department of General Surgery, The First Affiliated Hospital of Zhengzhou University, Zhengzhou, Henan, China

**Keywords:** liver abscess, invasive syndrome, multicenter study, predictive model, web-based calculator

## Abstract

**Background:**

Patients with liver abscess are at high risk of developing invasive K. pneumonia liver abscess syndrome (IKPLAS), which can worsen survival and quality of life. Early identification of high-risk patients is crucial. This study aimed to identify risk factors for IKPLAS and develop a predictive model to guide early intervention.

**Methods:**

We retrospectively collected data from 1,762 liver abscess patients at the First Hospital of Jilin University between 2015 and 2024. Patients were randomly divided into a training set and an internal validation set at a 7:3 ratio, and 203 patients from another hospital served as an external validation cohort. The SMOTE algorithm was applied to address data imbalance. Independent risk factors were identified using LASSO and logistic regression analyses, and the performance of different models was compared. Ultimately, a LASSO-based logistic regression model was used to construct a predictive nomogram. Model performance was comprehensively evaluated using the area under the receiver operating characteristic curve (AUC), decision curve analysis (DCA), clinical impact curve (CIC), and calibration curve. An online risk calculator was also developed for clinical use.

**Results:**

Among 1,965 patients (1,304 males, 661 females; mean age 58.96 ± 13.07 years), 548 (28.9%) developed IKPLAS. Independent risk factors included CRP (OR = 1.005, 95% CI: 1.003–1.007), PLT (OR = 0.995, 95% CI: 0.994–0.997), Prior biliary disease (OR = 1.137, 95% CI: 1.025–2.571), Fever (OR = 2.196, 95% CI: 1.292–3.824), Pleural effusion (OR = 7.355, 95% CI: 4.883–14.761), Ascites (OR = 8.786, 95% CI: 5.141–9.342), Broth culture (OR = 2.264, 95% CI: 1.186–3.371), DM (OR = 2.516, 95% CI: 1.757–3.63), and TBIL (OR = 1.006, 95% CI: 1.002–1.010). The nomogram achieved AUCs of 0.960, 0.920, and 0.892 in the training, internal, and external validation sets, respectively, with good calibration and clinical utility.

**Conclusion:**

We developed a nine-factor nomogram to predict individualized IKPLAS risk, demonstrating high discrimination and calibration, supporting early identification of high-risk patients and personalized management.

## Introduction

Liver abscess is a localized purulent inflammation of the liver caused by bacterial infection, with common symptoms including fever, upper abdominal pain, nausea, and weight loss (1). With the increasing prevalence of underlying diseases such as cirrhosis, biliary disorders, and diabetes, the incidence of liver abscess has shown a significant upward trend, especially in Asian regions such as China (2, 3). This trend is closely related to the high burden of liver disease in the region, as well as the distribution of medical resources and differences in early disease diagnosis levels. According to epidemiological studies, approximately 20% of liver abscess patients, due to delayed diagnosis and treatment, develop into Invasive K. pneumoniae liver abscess syndrome (IKPLAS), significantly increasing mortality rates (4). This not only imposes a heavy burden on the patients themselves but also places significant strain on public health systems (5).

IKPLAS, as a systemic disease caused by bacterial infection, is typically accompanied by multiple organ dysfunction (4, 6, 7). For liver abscess patients, the occurrence of IKPLAS not only complicates the treatment process but also has a profound impact on the clinical prognosis. Early diagnosis of IKPLAS is highly challenging, as conventional diagnostic methods, such as blood cultures, imaging examinations, and biochemical marker testing, often have significant limitations in sensitivity and specificity during the early stages of the disease (4, 8). Moreover, the clinical manifestations of IKPLAS greatly overlap with those of various other diseases, which can lead to misdiagnosis or missed diagnosis, thus increasing the difficulty of clinical management (9, 10).

In recent years, with the rapid development of predictive modeling and data mining technologies, an increasing number of studies have attempted to construct predictive models based on clinical and omics-related data to improve the early diagnostic accuracy of IKPLAS (8, 11, 12). These models can effectively integrate patients’ clinical characteristics, laboratory test results, and medical history, providing clinicians with more precise diagnostic tools. Although some predictive models have demonstrated certain predictive capabilities in specific populations, current research still faces several limitations. Firstly, many studies have relatively small sample sizes, which restrict the generalizability and accuracy of the models (11). Secondly, most existing models are derived from Western populations and lack validation in Chinese and other Asian populations, making their applicability to Chinese patients with liver abscess unclear (13). More importantly, many models have not been externally validated using independent datasets, thereby limiting their clinical applicability (8, 10, 11).

This study, based on a multi-center retrospective cohort dataset from China, aimed to identify risk factors associated with the development of IKPLAS in patients with liver abscess and to construct and validate an effective predictive model. In the training set, the Synthetic Minority Oversampling Technique (SMOTE) was applied to address data imbalance (14, 15). Subsequently, LASSO regression was employed to selectively screen variables and apply a penalty mechanism, thereby enhancing the stability and predictive accuracy of the model (16). The ultimate goal of this study was to develop a reliable early prediction tool to support clinical decision-making, optimize the management of patients with liver abscess, reduce the incidence of IKPLAS, and improve clinical outcomes.

## Materials and methods

### Study design and population

**FIGURE 1:**
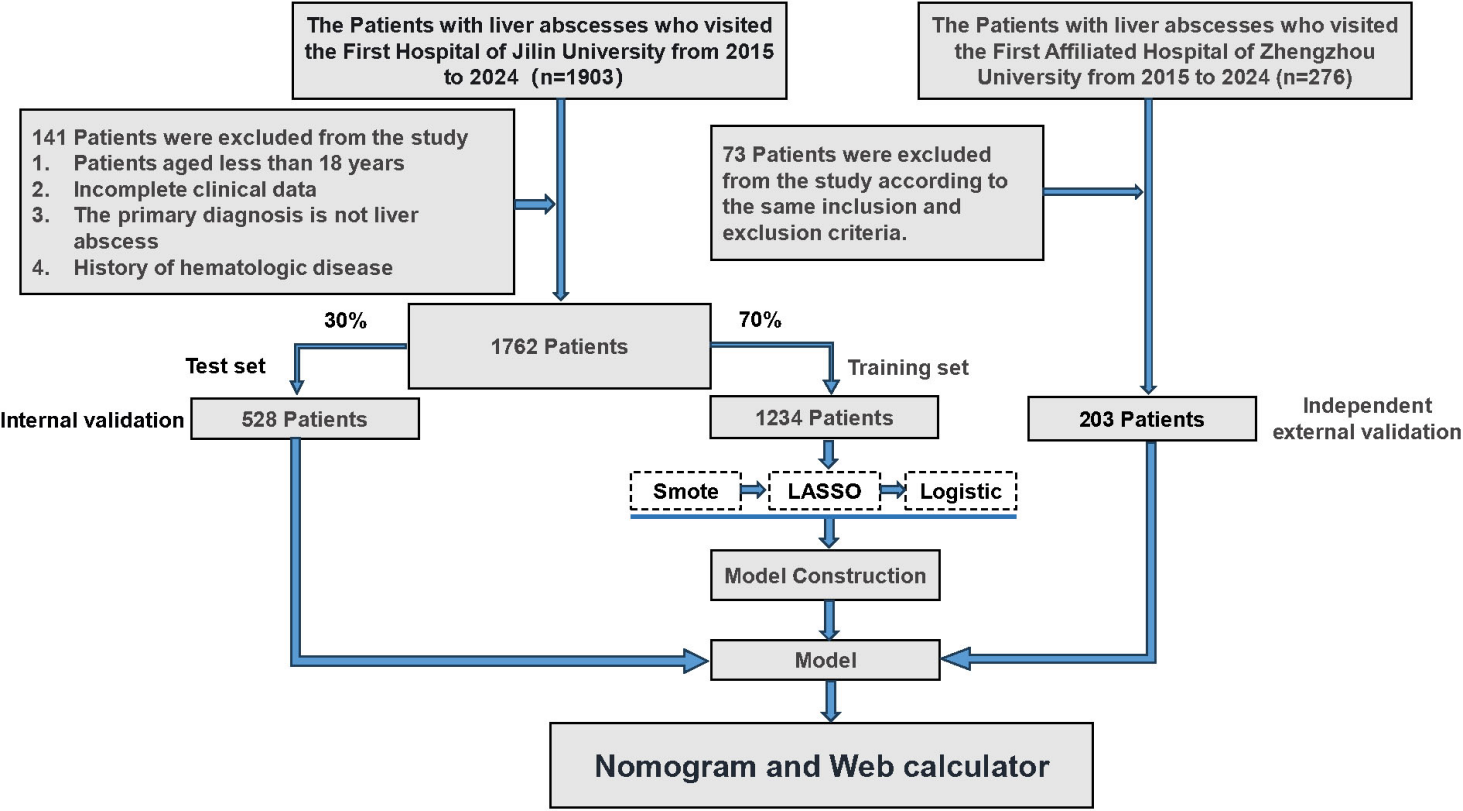
Flowchart illustrating the step-by-step process of patient selection from the liver disease database.

This study is a multi-center retrospective cohort study conducted in China, including patients diagnosed with Klebsiella pneumoniae liver abscess between January 2015 and December 2024. The exclusion criteria were as follows: (1) patients under 18 years of age; (2) incomplete clinical data, including medical history, laboratory test results, or imaging findings; (3) non-primary liver abscess infection sites; (4) a history of hematologic diseases. Patients with missing key clinical or laboratory variables were excluded from the analysis. To minimize the impact of incomplete data on model performance, we adopted a complete-case analysis approach (Figure 1). In this study, IKPLAS was defined as a confirmed diagnosis of K. pneumoniae liver abscess accompanied by metastatic infections, which may include, but are not limited to, abdominal infections, lung abscesses, endophthalmitis, meningitis, and necrotizing fasciitis. The diagnosis of IKPLAS was independently assessed by two senior clinicians and confirmed with the assistance of radiologists and laboratory specialists. A diagnosis was only established when both clinicians reached a consensus confirming the presence of IKPLAS. This study was approved by the Ethics Committee of the First Hospital of Jilin University and conducted in accordance with the Declaration of Helsinki (17). All data were collected and processed under strict privacy protection measures. As a retrospective study, all data were anonymized and no personal patient information was disclosed; therefore, informed consent was not required.

### Data collection

Data for this study were retrospectively collected from the electronic medical records system of the First Hospital of Jilin University and other participating hospitals. The clinical data involved included patients’ demographic information, laboratory test results, imaging findings, and other relevant clinical parameters. The specific data items are as follows:

Regarding demographic information, data collected included age, gender, body mass index (BMI), smoking history, drink consumption history, history of prior biliary surgery, history of diabetes mellitus (DM), history of hypertension, underlying heart disease, and kidney insufficiency. Laboratory tests included routine biochemical markers such as white blood cell count (WBC), hemoglobin (HB), platelet count (PLT), C-reactive protein (CRP), procalcitonin (PCT), and prothrombin time (PT). Additionally, liver function-related parameters were collected, including aspartate aminotransferase (AST), alanine aminotransferase (ALT), γ-glutamyl-transferase (γ-GT), alkaline phosphatase (ALP), albumin (ALB), total bilirubin (TBIL), and creatinine. Microbiological data included blood cultures and broth culture.

Regarding imaging findings, the abscess size, abscess count, and abscess location were collected. Furthermore, information on the presence of pleural effusion, ascites, and other relevant imaging and clinical examination data was recorded. The diagnosis of IKPLAS was also documented. Data collection for medical history and routine blood tests was performed on the day of admission, while abdominal ultrasound, chest color Doppler ultrasound, or lung CT scans were based on the first examination after admission. The distribution characteristics of all variables in this study are shown in Supplementary Figure 1.

### Variable selection

**FIGURE 2:**
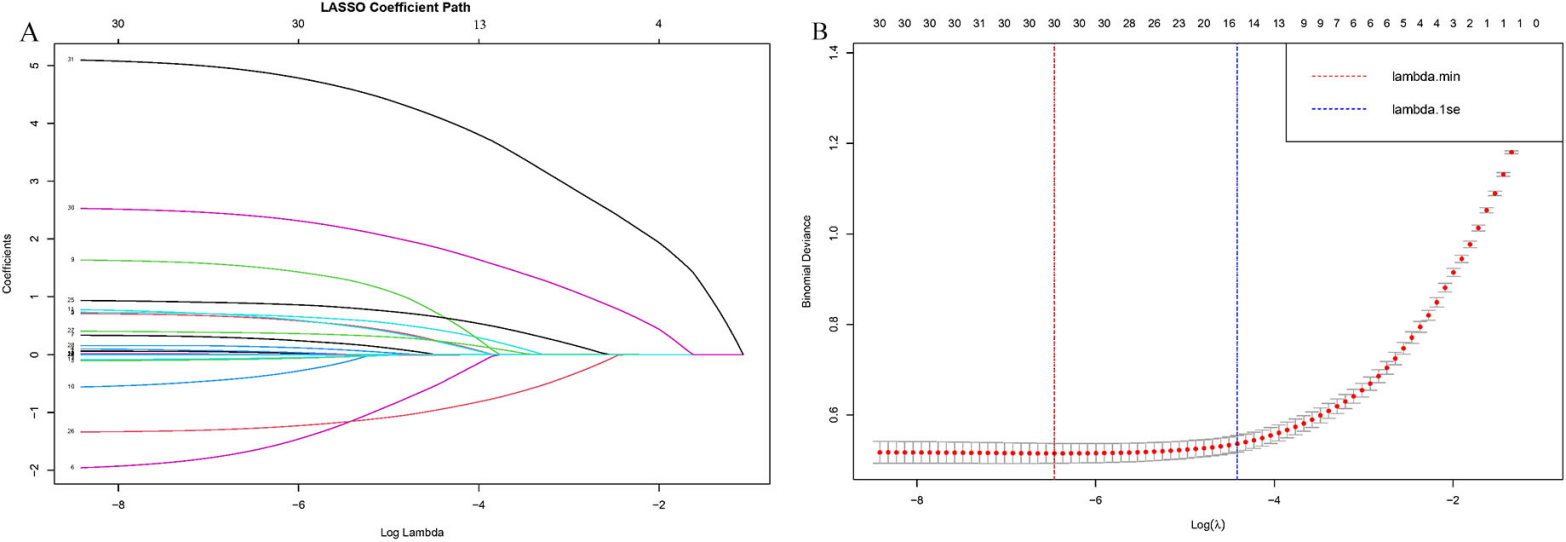
The selection process of the optimum value of the parameter λ in the LASSO regression model by cross-validation method. **(A)** LASSO coefficient profiles of the 30 risk factors, **(B)** cross-validation curve.

Based on existing literature and clinical expertise, 31 potential predictive variables were initially identified. In the training set, class imbalance was first addressed using the SMOTE algorithm. Subsequently, LASSO regression was applied to the oversampled training data to select the most relevant variables, resulting in the inclusion of 15 variables: DM, Drink, Prior history of biliary disease, Hypertension, Underlying heart disease, Fever, PLT, CRP, AST, TBIL, Creatinine, Broth Culture, Abscess Location, Pleural Effusion, and Ascites (Figure 2). These variables were then subjected to univariate logistic regression analysis, and those with *P* < 0.05 were further included in multivariate logistic regression analysis to identify independent risk factors for the development of IKPLAS in patients with liver abscess. Based on these independent risk factors, an individualized predictive model was constructed to accurately assess the risk of IKPLAS in liver abscess patients.

### Statistical analysis

Statistical analyses were performed using SPSS 26.0 and R 4.3.2. Among the 31 initially included variables, categorical variables were compared using the chi-square test, while continuous variables were presented as mean ± standard deviation (Mean ± SD) and compared using independent-sample *t*-tests. In the training set, class imbalance was first addressed using the SMOTE algorithm from the UBL package in R. Given the large number of potential predictors, LASSO regression was subsequently applied to the oversampled training data to select the optimal variables, thereby reducing multicollinearity and preventing model overfitting. Univariate and multivariate logistic regression analyses were then performed to identify independent risk factors associated with the development of IKPLAS. Based on these independent risk factors, the LASSO-based logistic regression model was systematically compared with several commonly used machine learning algorithms, including XGBoost and random forest. The results demonstrated that all three models achieved satisfactory diagnostic performance in the training set, with AUC values exceeding 0.90. In the validation set, the AUC values also exceeded 0.85, indicating good discriminative ability for all models (Supplementary Figure 2). Considering the balance between model performance and interpretability, we ultimately selected the LASSO-based logistic regression model. Unlike “black-box” algorithms, LASSO regression allows for direct variable selection and provides clear coefficients indicating the direction and magnitude of each variable’s effect on the outcome, which is particularly important for clinical interpretation and decision-making. Using these independent risk factors, an individualized early-prediction nomogram was constructed with the “rms” package in R, and an interactive online nomogram was developed using the Shiny package. Detailed R code is provided in Supplementary material. Model performance was comprehensively evaluated using AUC, DCA, CIC, and calibration curves to assess discrimination, clinical utility, and calibration. All statistical tests were two-sided, with *P* < 0.05 considered statistically significant.

## Results

### Clinical characteristics of liver abscess patients

This study included 1,762 patients from the derivation cohort at the First Hospital of Jilin University and 203 patients from the external validation cohort at the First Affiliated Hospital of Zhengzhou University. In the derivation cohort, 501 patients (28.4%) were diagnosed with IKPLAS and were randomly divided into a training set and an internal validation set at a ratio of 7:3. In the external validation cohort, 47 patients (23.15%) developed IKPLAS. There was no statistically significant difference in the incidence of IKPLAS between the two cohorts (*P* = 0.056) (Table 1).

**TABLE 1:** Comparison of baseline characteristics of liver abscess patients between two different hospitals.

N (%)	Category	Total (*N* = 1965)	Derivation cohort (*N* = 1762)	External validation cohort (*N* = 203)	*P-*value
**Demographic data**					
Age, years		58.96 (13.07)	59.02 (13.08)	58.37 (13.28)	0.498
Gender (%)	Male	1304 (66.36)	1159 (65.78)	145 (71.43)	0.125
	Female	661 (33.64)	603 (34.22)	58 (28.57)	
BMI (%)	Under weight	706 (35.93)	637 (36.15)	69 (33.99)	0.943
	Normal weight	401 (20.41)	359 (20.37)	42 (20.69)	
	Overweight	437 (22.24)	390 (22.13)	47 (23.15)	
	Obesity	421 (21.42)	376 (21.34)	45 (22.17)	
Smoke (%)	No	1049 (53.38)	927 (52.61)	122 (60.10)	0.051
	Yes	916 (46.62)	835 (47.39)	81 (39.90)	
Drink (%)	No	1004 (51.09)	904 (51.31)	100 (49.26)	0.633
	Yes	961 (48.91)	858 (48.69)	103 (50.74)	
Prior history of biliary disease (%)	No	1898 (96.59)	1702 (96.59)	196 (96.55)	1.00
	Yes	67 (3.41)	60 (3.41)	7 (3.45)	
DM (%)	No	1086 (55.27)	944 (53.58)	142 (69.95)	< 0.001
	Yes	879 (44.73)	818 (46.42)	61 (30.05)	
Hypertension (%)	No	1736 (88.35)	1552 (88.08)	184 (90.64)	0.337
	Yes	229 (11.65)	210 (11.92)	19 (9.36)	
Underlying heart disease (%)	No	1922 (97.81)	1720 (97.62)	202 (99.51)	0.136
	Yes	43 (2.19)	42 (2.38)	1 (0.49)	
Kidney insufficiency (%)	No	1893 (96.34)	1701 (96.54)	192 (94.58)	0.227
	Yes	72 (3.66)	61 (3.46)	11 (5.42)	
Fever (%)	No	338 (17.20)	312 (17.71)	26 (12.81)	0.098
	Yes	1627 (82.80)	1450 (82.29)	177 (87.19)	
**Laboratory data**					
WBC [mean (SD)]		12.54 (6.16)	12.51 (6.07)	12.77 (6.92)	0.561
HB [mean (SD)]		122.73 (20.17)	122.79 (20.46)	122.22 (17.50)	0.704
PLT [mean (SD)]		215.65 (133.50)	214.64 (132.64)	224.37 (140.82)	0.326
CRP [mean (SD)]		130.24 (112.68)	133.41 (113.64)	102.67 (100.02)	< 0.001
PCT [mean (SD)]		22.68 (27.51)	22.84 (27.92)	21.35 (23.68)	0.465
PT [mean (SD)]		13.37 (2.44)	13.37 (2.47)	13.40 (2.24)	0.849
AST [mean (SD)]		83.59 (176.21)	83.43 (173.57)	85.03 (198.14)	0.902
ALT [mean (SD)]		84.17 (121.77)	83.85 (119.40)	86.93 (140.96)	0.733
Y-GT [mean (SD)]		192.38 (185.58)	192.60 (186.80)	190.42 (175.08)	0.874
ALP [mean (SD)]		188.89 (145.15)	191.33 (148.35)	167.67 (111.69)	0.028
ALB [mean (SD)]		30.73 (11.00)	30.89 (11.44)	29.39 (5.62)	0.066
TBIL [mean (SD)]		29.66 (37.86)	30.30 (39.23)	24.11 (21.91)	0.027
Creatinine [mean (SD)]		85.31 (67.29)	86.31 (68.47)	76.63 (55.37)	0.053
Blood culture (%)	No	1393 (70.89)	1251 (71.00)	142 (69.95)	0.818
	Yes	572 (29.11)	511 (29.00)	61 (30.05)	
Broth culture (%)	No	1132 (57.6)	1050 (59.6)	82 (40.39)	< 0.001
	Yes	833 (42.4)	712 (40.4)	121 (59.61)	
**Imaging data**					
Abscess size [mean (SD)]		57.95 (31.95)	58.07 (31.86)	56.82 (32.79)	0.599
Single abscess (%)	No	1373 (69.87)	1226 (69.58)	147 (72.41)	0.452
	Yes	592 (30.13)	536 (30.42)	56 (27.59)	
Abscess location (%)	Right side	1131 (57.56)	1014 (57.55)	117 (57.64)	0.397
	Left side	476 (24.22)	421 (23.89)	55 (27.09)	
	Bilateral	358 (18.22)	327 (18.56)	31 (15.27)	
Pleural effusion (%)	No	1675 (85.24)	1494 (84.79)	181 (89.16)	0.119
	Yes	290 (14.76)	268 (15.21)	22 (10.84)	
Ascites (%)	No	1374 (88.60)	1194 (67.8)	180 (88.67)	1.00
	Yes	591 (11.40)	568 (32.2)	23 (11.33)	
IKPLAS (%)	No	1427 (73.1)	1261 (71.6)	156 (86.70)	0.056
	Yes	548 (28.9)	501 (28.40)	47 (23.15)	

BMI, Body Mass Index; DM, Diabetes Mellitus; WBC, White Blood Cell; Hb, Hemoglobin; PLT, Platelet; CRP, C-Reactive Protein; PCT, Procalcitonin; PT, Prothrombin Time; AST, Aspartate Aminotransferase; ALT, Alanine Aminotransferase; γ-GT, Gamma-Glutamyl Transferase; ALP, Alkaline Phosphatase; ALB, Albumin; TBIL, Total Bilirubin; IKPLAS, Invasive K. pneumoniae liver abscesses syndrome.

In the derivation cohort, we compared the clinical characteristics and laboratory parameters between patients with and without IKPLAS (Table 2). The results showed that patients with IKPLAS differed significantly in several clinical and laboratory variables. Specifically, the proportions of diabetes, hypertension, and underlying heart disease were higher among patients with IKPLAS, and fever was more often the initial symptom (all *P* < 0.05).

**TABLE 2:** Baseline characteristics between liver abscesses patients with and without invasive syndrome.

N (%)	Category	Total (*N* = 1762)	Without invasive syndrome (*N* = 1505)	With invasive syndrome (*N* = 257)	*P*-value
**Demographic data**					
Age, years		59.02 (13.07)	58.98 (13.14)	59.25 (12.70)	0.761
Gender (%)	Male	1159 (65.78)	985 (65.45)	174 (67.70)	0.527
	Female	603 (34.22)	520 (34.55)	83 (32.30)	
BMI (%)	Under weight	637 (36.15)	542 (36.01)	95 (36.96)	0.642
	Normal weight	359 (20.37)	309 (20.53)	50 (19.46)	
	Over weight	390 (22.13)	327 (21.73)	63 (24.51)	
	Obesity	376 (21.34)	327 (21.73)	49 (19.07)	
Smoke (%)	No	927 (52.61)	798 (53.02)	129 (50.19)	0.440
	Yes	835 (47.39)	707 (46.98)	128 (49.81)	
Drink (%)	No	904 (51.31)	786 (52.23)	118 (45.91)	0.071
	Yes	858 (48.69)	719 (47.77)	139 (54.09)	
Prior history of biliary disease (%)	No	1702 (96.59)	1449 (96.28)	253 (98.44)	0.114
	Yes	60 (3.41)	56 (3.72)	4 (1.56)	
DM (%)	No	944 (53.58)	860 (57.14)	84 (32.68)	< 0.001
	Yes	818 (46.42)	645 (42.86)	173 (67.32)	
Hypertension (%)	No	1552 (88.08)	1347 (89.50)	205 (79.77)	< 0.001
	Yes	210 (11.92)	158 (10.50)	52 (20.23)	
Underlying heart disease (%)	No	1720 (97.62)	1475 (98.01)	245 (95.33)	0.017
	Yes	42 (2.38)	30 (1.99)	12 (4.67)	
Kidney insufficiency (%)	No	1701 (96.54)	1454 (96.61)	247 (96.11)	0.824
	Yes	61 (3.46)	51 (3.39)	10 (3.89)	
Fever (%)	No	312 (17.71)	283 (18.80)	29 (11.28)	0.005
	Yes	1450 (82.29)	1222 (81.20)	228 (88.72)	
Laboratory data					
WBC [mean (SD)]		12.51 (6.07)	12.398 (5.65)	13.15 (8.05)	0.065
HB [mean (SD)]		122.79 (20.46)	122.21 (20.04)	126.18 (22.50)	0.004
PLT [mean (SD)]		214.64 (132.64)	230.10 (130.82)	124.10 (104.14)	< 0.001
CRP [mean (SD)]		133.41(113.64)	121.54 (111.00)	202.92 (103.76)	< 0.001
PCT [mean (SD)]		22.84 (27.92)	20.58 (26.27)	36.04 (33.21)	< 0.001
PT [mean (SD)]		13.37 (2.47)	13.29 (2.44)	13.79 (2.60)	0.003
AST [mean (SD)]		83.43 (173.57)	70.15 (112.91)	161.16 (353.88)	< 0.001
ALT [mean (SD)]		83.85 (119.40)	76.15 (88.98)	128.89 (221.71)	< 0.001
γ-GT [mean (SD)]		192.60 (186.80)	193.93 (191.94)	184.84 (153.35)	0.471
ALP [mean (SD)]		191.33 (148.35)	190.69 (145.35)	195.09(165.10)	0.660
ALB [mean (SD)]		30.89 (11.44)	31.23 (12.11)	28.88 (5.83)	0.002
TB [mean (SD)]		30.30 (39.23)	27.82 (35.30)	44.85 (54.93)	< 0.001
Creatinine [mean (SD)]		86.31 (68.47)	80.80 (63.24)	118.53 (86.79)	< 0.001
Blood culture (%)	No	1251 (71.00)	1088 (72.29)	163 (63.42)	0.005
	Yes	511 (29.00)	417 (27.71)	94 (36.58)	
Broth culture (%)	No	1050 (59.6)	901 (59.9)	149 (57.98)	< 0.001
	Yes	712 (40.4)	604 (40.1)	108 (42.02)	
**Imaging data**					
Abscess size [mean (SD)]		58.07 (31.86)	58.18 (31.88)	57.48 (31.80)	0.744
Single abscess (%)	No	1226 (69.58)	1053 (69.97)	173 (67.32)	0.435
	Yes	536 (30.42)	452 (30.03)	84 (32.68)	
Abscess location (%)	Right side	1014 (57.55)	873 (58.01)	141 (54.86)	< 0.001
	Left side	421 (23.89)	376 (24.98)	45 (17.51)	
	Bilateral	327 (18.56)	256 (17.01)	71 (27.63)	
Pleural effusion (%)	No	1194 (67.8)	1078 (59.3)	116 (45.14)	< 0.001
	Yes	568 (32.2)	427 (40.7)	141 (54.86)	
Ascites (%)	No	1261 (71.6)	1160 (77.1)	101 (39.30)	< 0.001
	Yes	501 (28.4)	345 (22.9)	156 (60.70)	

BMI, Body Mass Index; DM, Diabetes Mellitus; WBC, White Blood Cell; Hb, Hemoglobin; PLT, Platelet; CRP, C-Reactive Protein; PCT, Procalcitonin; PT, Prothrombin Time; AST, Aspartate Aminotransferase; ALT, Alanine Aminotransferase; γ-GT, Gamma-Glutamyl Transferase; ALP, Alkaline Phosphatase; ALB, Albumin; TBIL, Total Bilirubin.

Regarding laboratory findings, patients with IKPLAS had significantly higher levels of Hb, CRP, PT, and PCT, along with lower PLT. In terms of liver function, patients with IKPLAS exhibited higher levels of AST, ALT, and TBIL, but lower ALB levels. For renal function, creatinine levels were elevated in the IKPLAS group.

Microbiological examination revealed that the positive rates of blood culture and pus culture were significantly higher in the IKPLAS group (36.58 and 42.02%, respectively) compared with the non-IKPLAS group (27.71 and 40.10%, respectively). Imaging findings showed that patients with IKPLAS were more likely to present with bilobar liver abscesses, and the incidences of pleural effusion and ascites were significantly higher than in non-IKPLAS patients (all *P* < 0.05).

### Identifying independent risk factors for the development of invasive syndrome in liver abscess

In this study, the SMOTE algorithm was first applied to the training set to address the issue of class imbalance, resulting in a balanced ratio of positive to negative samples of 1:1 (Supplementary Figure 3). Subsequently, to reduce the dimensionality of the oversampled dataset and to identify key feature variables associated with the development of IKPLAS in liver abscess patients, LASSO regression analysis was performed for variable selection.

The initially included variables were: gender (male or female), age, BMI, Smoke (yes or no), Drink (yes or no), Prior history of biliary disease (yes or no), DM (yes or no), Hypertension (yes or no), Underlying heart disease (yes or no), Renal insufficiency (yes or no), Fever (yes or no), WBC, HB, PLT, CRP, PCT, PT, AST, ALT, γ-GT, ALP, ALB, TBIL, Creatinine, Blood culture (yes or no), Broth culture (yes or no), Abscess location, Single abscess (yes or no), Abscess diameter, Pleural effusion (yes or no), and Ascites (yes or no).

The LASSO regression results identified 15 potential variables associated with IKPLAS, including: DM, Drink, Prior history of biliary disease, Hypertension, Underlying heart disease, Fever, PLT, CRP, AST, TBIL, Creatinine, Broth culture, Abscess location, Pleural effusion, and Ascites (Figure 2).

Subsequently, these variables were incorporated into univariate logistic regression analysis, with a threshold of *P* < 0.05 used to identify variables significantly associated with IKPLAS. The analysis revealed that DM, Drink, Prior history of biliary disease, Hypertension, Underlying heart disease, Fever, PLT, CRP, AST, TBIL, Creatinine, Broth culture, Pleural effusion, and Ascites were all correlated with the occurrence of IKPLAS in liver abscess patients.

Finally, variables with statistical significance in the univariate analysis were included in multivariate logistic regression analysis. The results indicated that CRP (OR = 1.005, 95% CI: 1.003–1.007), PLT (OR = 0.995, 95% CI: 0.994–0.997), Prior biliary disease (OR = 1.137, 95% CI: 1.025–2.571), Fever (OR = 2.196, 95% CI: 1.292–3.824), Pleural effusion (OR = 7.355, 95% CI: 4.883–14.761), Ascites (OR = 8.786, 95% CI: 5.141–9.342), Broth culture (OR = 2.264, 95% CI: 1.186–3.371), DM (OR = 2.516, 95% CI: 1.757–3.63), and TBIL (OR = 1.006, 95% CI: 1.002–1.010) were independent risk factors for the development of IKPLAS in patients with liver abscess (Table 3).

**TABLE 3:** Univariate and multivariate logistic regression analysis of Invasive Syndrome after LASSO regression in the training cohort.

N (%)	Category	Univariable OR (95%CI)	*P-*value	Multivariable OR (95%CI)	*P-*value
**Demographic data**					
DM (%)	No	Reference	< 0.001	Reference	< 0.001
	Yes	3.332 (2.2661–4.389)		2.516 (1.757–3.63)	
Drink (%)	No	Reference	< 0.001		
	Yes	1.570 (1.304–1.891)			
Prior history of biliary disease (%)	No	Reference	< 0.001	Reference	0.013
	Yes	1.198 (1.087–1.400)		1.137 (1.025–1.571)	
Hypertension (%)	No	Reference	< 0.001	Reference	
	Yes	2.276 (1.737–2.997)			
Underlying heart disease (%)	No	Reference	0.02	Reference	
	Yes	2.719 (1.530–5.032)			
Fever (%)	No	Reference	0.012	Reference	0.004
	Yes	2.226 (1.714–2.906)		2.196 (1.292–3.824)	
**Laboratory data**					
PLT [mean (SD)]		0.993 (0.992–0.995)	< 0.001	0.995 (0.994–0.997)	< 0.001
CRP [mean (SD)]		1.008 (1.007–1.008)	< 0.001	1.005 (1.003–1.007)	< 0.001
AST [mean (SD)])		1.004 (1.003–1.005)	0.014		
TBIL [mean (SD)]		1.011 (1.008–1.015)	< 0.001	1.006 (1.002–1.010)	0.002
Creatinine [mean (SD)]		1.009 (1.007–1.011)	0.032		
Broth culture (%)	No	Reference	< 0.001	Reference	0.024
	Yes	0.322 (0.266–0.389)		2.264 (1.186–3.371)	
**Imaging data**					
Abscess location (%)	No	Reference	0.052	Reference	
	Yes	1.12 (0.998–1.256)			
Pleural effusion (%)	No	Reference	< 0.001	Reference	< 0.001
	Yes	7.645 (6.737–9.914)		7.355 (4.883–14.761)	
Ascites (%)	No	Reference	< 0.001	Reference	< 0.001
	Yes	9.243 (6.193–10.761)		8.786 (5.141–9.342)	

DM, Diabetes Mellitus; PLT, Platelet; CRP, C-Reactive Protein; AST, Aspartate Aminotransferase; TBIL, Total Bilirubin; OR: Odds Ratio; CI: Confidence Interval.

### Model development and validation

**FIGURE 3:**
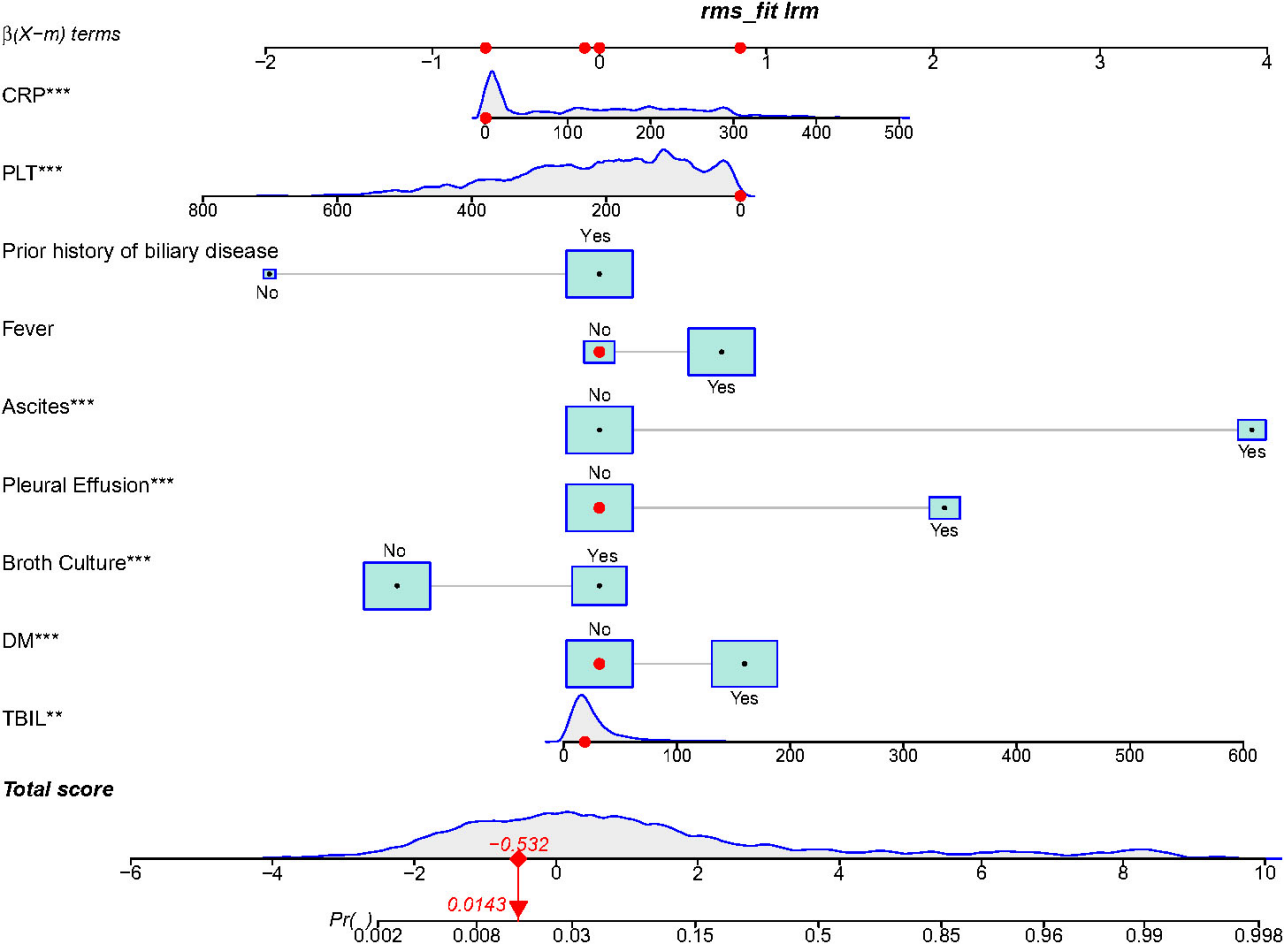
Nomogram predicting the occurrence of invasive syndrome in patients with pyogenic liver abscess. **p ¡ 0.01, ***p ¡ 0.001.

**FIGURE 4:**
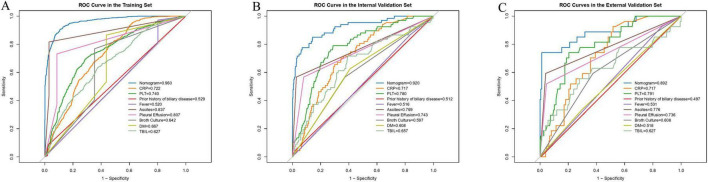
ROC curves in **(A–C)** illustrate the predictive performance of the model for the occurrence of invasive syndrome in patients with pyogenic liver abscess. **(A)** Represents the training set, **(B)** represents the internal test set, and **(C)** represents the external test set. The ROC curve provides a graphical representation of the true positive rate against the false positive rate, offering insights into the accuracy and discriminative ability of the nomogram.

Based on the independent risk factors identified above, we developed an individualized risk prediction model to accurately estimate the risk of IKPLAS in liver abscess patients (Figure 3). To better integrate these predictive factors, a nomogram was constructed. The performance of the model was evaluated by plotting ROC curves in the training set, internal validation set, and external validation set, with AUC values of 0.960, 0.920, and 0.892, respectively, all of which were significantly higher than the AUC values of any individual independent predictor (Table 4; Figure 4). Furthermore, to assess the relative advantage of the model, we compared the LASSO-based logistic regression model with commonly used machine learning algorithms, including XGBoost and random forest. The results showed that XGBoost and random forest also performed well in both the training and validation sets. However, considering both model discrimination and clinical interpretability, we ultimately selected the LASSO-based logistic regression model as the final modeling approach for this study (Supplementary Figure 2).

**TABLE 4:** AUC of training set and validation set.

Variable	Training group		Internal validation		External validation	
	AUC	95%CI	AUC	95%CI	AUC	95%CI
Nomogram	0.960	0.954–0.968	0.920	0.881–0.960	0.892	0.813–0.971
DM	0.667	0.619–0.664	0.608	0.546–0.671	0.518	0.496–0.759
Prior history of biliary disease	0.529	0.520–0.538	0.512	0.487–0.538	0.497	0.492–0.503
Fever	0.520	0.503–0.537	0.516	0.472–0.561	0.531	0.475–0.588
PLT	0.740	0.719–0.762	0.780	0.725–0.837	0.791	0.705–0.878
CRP	0.722	0.701–0.745	0.717	0.661–0.775	0.717	0.634–0.801
TBIL	0.627	0.604–0.691	0.657	0.585–0.731	0.627	0.496–0.759
Broth culture	0.642	0.620–0.666	0.597	0.533–0.661	0.608	0.508–0.710
Pleural effusion	0.807	0.789–0.824	0.743	0.682–0.805	0.736	0.639–0.834
Ascites	0.834	0.814–0.901	0.769	0.709–0.830	0.776	0.681–0.872

DM, Diabetes Mellitus; PLT, Platelet; CRP, C-Reactive Protein; TBIL, Total Bilirubin; OR: Odds Ratio; CI: Confidence Interval; AUC, Area Under the Curve.

### Decision curve and clinical impact curve analysis

**FIGURE 5:**
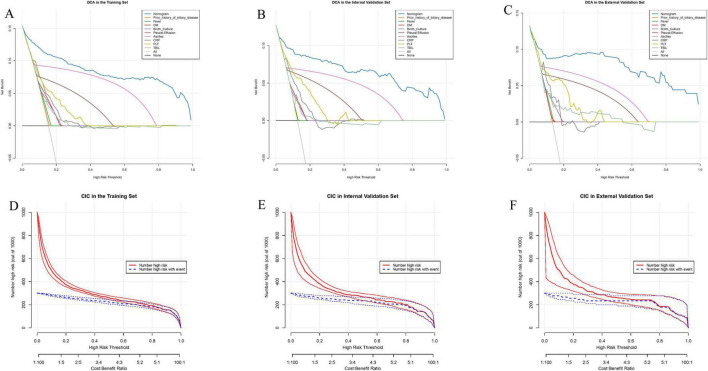
Decision curve analysis (DCA) and clinical impact curve (CIC) illustrate the clinical utility of the nomogram. The net benefit of using the nomogram to predict the occurrence of invasive syndrome in patients with pyogenic liver abscess was evaluated. **(A,D)** Represent the predictions in the training set, **(B,E)** represent the internal test set, and **(C,F)** represent the external test set.

To assess the clinical applicability of the predictive nomogram, we performed DCA analysis and CIC analysis. The results of the DCA analysis showed that the predictive model provided a higher net benefit across a wide range of threshold probabilities in all datasets, outperforming the traditional “treat all” or “treat none” strategies. This indicates that the model can assist clinicians in optimizing treatment decisions and improving the accuracy of prognostic assessments (Figures 5A–C). The CIC analysis further demonstrated the model’s practical utility in guiding treatment decisions in clinical practice (Figures 5D–F). In conclusion, both DCA and CIC analyses confirmed the significant clinical value of the predictive nomogram, particularly in enhancing decision-making quality and patient management.

### Calibration curve analysis

**FIGURE 6:**
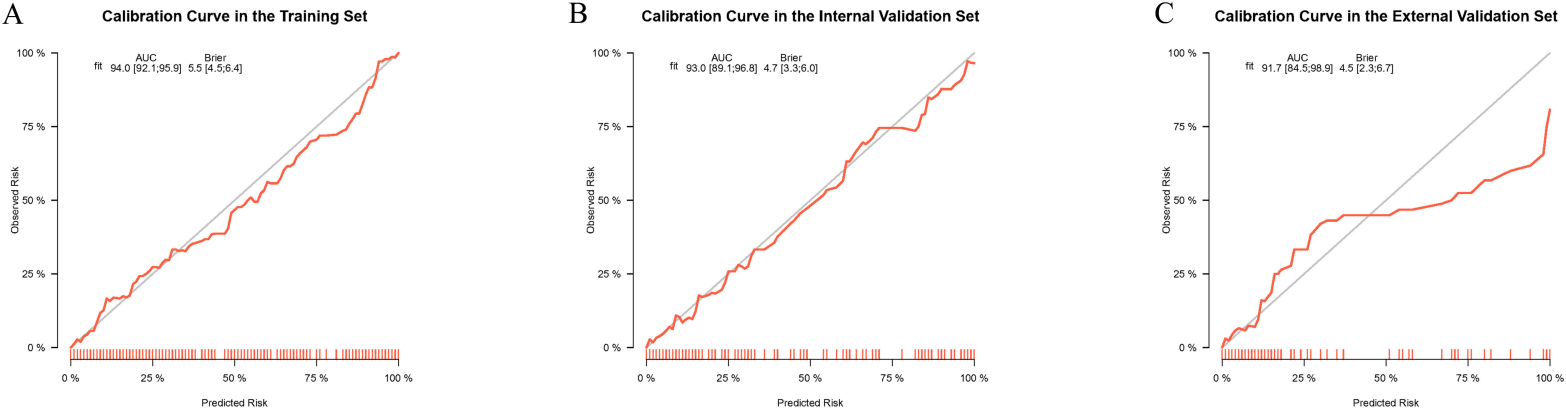
Calibration curves for evaluating the predictive performance of the model for the occurrence of invasive syndrome in patients with pyogenic liver abscess in the training set **(A)**, internal test set **(B)**, and external test set **(C)**. The calibration curves visually represent the calibration of the predictive model.

To assess the calibration ability of the prediction nomogram, we plotted calibration curves. The calibration curves demonstrated that the predicted probabilities closely matched the actual occurrence probabilities in the training set, internal validation set, and external validation set, indicating that the model has good calibration performance. Specifically, the calibration curves closely followed the ideal 45-degree line, suggesting that the model can accurately predict the risk of IKPLAS in liver abscess patients (Figure 6). These results further support the stability and reliability of the prediction model across different datasets.

### Establishment of the network computation model

**FIGURE 7:**
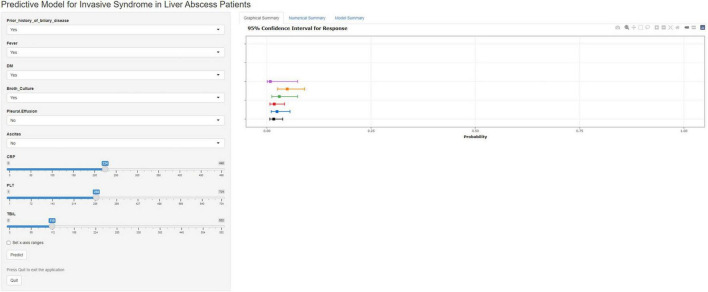
A web calculator developed based on nomogram.

To facilitate practical use by clinicians, we developed an online calculator that allows for the rapid prediction of the risk of IKPLAS in liver abscess patients based on their clinical and laboratory data. This online tool incorporates independent risk factors, including CRP, PLT, Prior biliary disease, Fever, Pleural effusion, Ascites, Broth culture, DM, and TBIL (Figure 7).^1^

## Discussion

Liver abscess is a purulent liver disease caused by various bacterial infections, often accompanied by severe clinical complications, such as IKPLAS (4). IKPLAS not only leads to multiple organ failure but also increases the mortality risk of patients (7). Early identification and intervention are key factors in improving the prognosis of patients with IKPLAS. Therefore, developing an effective predictive model for the occurrence of IKPLAS in liver abscess patients holds significant clinical value. This study, based on multicenter clinical data from China, aims to construct a nomogram model to predict the risk of developing IKPLAS in hospitalized patients with liver abscess. The model is developed using clinical variables through LASSO regression analysis, as well as univariate and multivariate logistic regression analyses.

Previous studies have reported that more than 60% of patients with liver abscess have DM, and elevated blood glucose levels at admission are significantly associated with poor outcomes, which is consistent with our findings (2, 18). In our study, the proportion of diabetic patients was significantly higher in those who developed IKPLAS. We speculate that impaired immune function and poor glycemic control in diabetic patients increase their susceptibility to infection and systemic inflammatory responses, thereby elevating the risk of sepsis and other IKPLAS-related complications (19, 20). Moreover, impaired phagocytosis of K1/K2 Klebsiella pneumoniae and increased vascular permeability in diabetic patients may further facilitate bacterial invasion (21–23)

PLT has been widely recognized as an important predictor of infectious diseases. Abnormal platelet levels often reflect inflammatory activation and coagulation dysfunction, serving as potential biomarkers of infection (24–26). A recent 2025 study demonstrated that PLT and other coagulation-related indices have good predictive value for assessing disease severity and prognosis in elderly sepsis patients, showing high sensitivity and specificity (25). Furthermore, Mendelian randomization studies have revealed a causal relationship between low platelet counts and increased sepsis risk, with thrombocytopenia being closely associated with poor outcomes (27, 28). In addition, immune dysregulation in sepsis may suppress bone marrow function, the primary site of platelet production, leading to further thrombocytopenia (29, 30).

CRP and TBIL are commonly used clinical indicators of inflammation and liver function. Numerous studies have shown that CRP levels are significantly elevated in infectious diseases, including liver abscess, and that persistent CRP elevation is often associated with poor prognosis (31, 32). A 2025 prospective cohort study demonstrated that the trend of CRP changes during hospitalization has good discriminatory ability in predicting mortality among sepsis patients (33). Our study further confirmed that CRP is a sensitive biomarker for predicting IKPLAS in liver abscess patients, with elevated levels indicating worse clinical outcomes, while a decline in CRP suggests better prognosis. Moreover, TBIL level on admission was also included in our predictive model. Elevated bilirubin levels have been frequently observed not only in patients with liver cirrhosis but also in critically ill patients with hypoxia-induced hepatocellular injury or sepsis (34, 35). Hyperbilirubinemia is strongly associated with poor prognosis in both liver cirrhosis and critically ill patients, suggesting that bilirubin elevation may serve as an early warning indicator of invasive complications in liver abscess patients (36).

In our study, a history of biliary disease was identified as an independent risk factor for IKPLAS (37, 38). This association may be related to anatomical or functional abnormalities of the biliary system, which promote bacterial translocation; colonization by multidrug-resistant organisms due to prior antibiotic exposure; and impaired hepatic immune defense following biliary surgery or interventional procedures. Additionally, chronic biliary inflammation can cause immune dysregulation and reduce hepatic reserve, both of which may exacerbate systemic inflammatory responses when infection occurs.

Positive broth culture results play a crucial role in identifying causative pathogens and guiding antibiotic therapy. Enterobacteriaceae, Enterococcus, and Streptococcus species are among the most common pathogens associated with liver abscess, while anaerobic bacteria from the gastrointestinal or biliary tract can promote infection spread, abscess formation, and sepsis (39, 40). Pseudomonas aeruginosa, characterized by strong resistance and high virulence, may cause severe invasive infections, particularly in immunocompromised patients (41). Unfortunately, our study did not further analyze the relationship between specific pathogens and IKPLAS risk.

The presence of pleural effusion and ascites often indicates infection spread or complications associated with liver abscess, both of which were significantly correlated with poor outcomes (42, 43). In our study. pleural effusion is commonly associated with sepsis or multiple organ dysfunction syndrome, possibly due to systemic inflammation and fluid redistribution, leading to respiratory failure (43). Ascites may suggest abscess rupture or worsening hepatic injury, potentially caused by abscess extension or biliary damage, which increase intra-abdominal pressure and exacerbate hepatic dysfunction (44). These complications markedly increase the risk of IKPLAS, especially in immunocompromised patients, where pleural effusion and ascites often serve as early warning signs of sepsis and multiorgan failure. Therefore, early recognition and timely management of these complications are essential for improving patient prognosis.

In this study, we focused on common clinical parameters, all of which have been shown to be closely associated with the occurrence of invasive disease in patients with liver abscess. We integrated multiple key predictive variables to develop a personalized model for assessing the risk of severe IKPLAS in liver abscess patients. During model development, the predictive potential of each variable was maximized, and the model demonstrated good predictive performance after derivation and external validation. To facilitate clinical application, we also developed a convenient web-based calculator that allows clinicians to quickly and accurately assess a patient’s risk at the bedside and adjust treatment plans accordingly, thereby improving patient outcomes. Compared with previous studies, our research offers several innovations and advantages. First, this study is a large-sample, multi-center retrospective cohort study, including liver abscess patients from different regions, which provides high representativeness and a certain degree of generalizability. Second, there is currently no widely recognized risk prediction model for IKPLAS in liver abscess patients. By integrating multiple clinical indicators, our model provides clinicians with a personalized risk assessment tool. Finally, the web-based calculator developed in this study offers a convenient and intuitive approach for risk prediction, making the model more accessible and practical, which is especially valuable for primary hospitals and less experienced clinicians.

However, this study has several limitations. First, although we used a relatively large sample, the data were derived from a retrospective cohort, which may introduce selection bias, and some potential influencing factors were not included in the analysis—for example, whether the abscess was gas-producing, the specific pathogens involved, and detailed antibiotic regimens—factors that could significantly affect patient prognosis. Second, the study only included data from two large tertiary hospitals in China, which may limit the generalizability of the model. In addition, some variables may be subject to measurement errors or incomplete documentation, particularly certain clinical and imaging indicators, which could affect the accuracy of the model. Furthermore, the applicability of the model in extreme values or specific subgroups (e.g., very elderly patients or those with severe underlying conditions) remains unclear. Finally, due to limitations in follow-up duration and outcome assessment, the model’s ability to predict long-term prognosis requires further validation. Therefore, in the future, we plan to conduct multicenter prospective studies based on our center, in collaboration with additional domestic and international institutions, to further validate and optimize the model’s applicability, external generalizability, and long-term predictive performance.

## Conclusion

This study identified CRP, PLT, Prior biliary disease, Fever, Pleural effusion, Ascites, Broth culture, DM, and TBIL as significant predictors of the occurrence of IKPLAS in liver abscess patients. Based on these key factors, we developed a personalized risk assessment model aimed at evaluating the risk of progression to IKPLAS in liver abscess patients. To enhance the model’s applicability, we also designed a convenient web-based calculator to facilitate easy use by clinicians (see text footnote 1). The development of this tool is intended to provide valuable support for clinical practice, helping healthcare professionals better assess and manage the condition of liver abscess patients.

## Data Availability

The raw data supporting the conclusions of this article will be made available by the authors, without undue reservation.

## Footnotes

1
https://shaoguangzhao0827shao.shinyapps.io/ICLAModel/

## References

[1] Hullahalli K Dailey KG Hasegawa Y Torres E Suzuki M Zhang H Genetic and immune determinants of *E. coli* liver abscess formation. *Proc Natl Acad Sci U S A*. (2023) 120:e2310053120. 10.1073/pnas.2310053120 38096412 PMC10743367

[2] Thomsen RW Jepsen P Sørensen HT. Diabetes mellitus and pyogenic liver abscess: risk and prognosis. *Clin Infect Dis*. (2007) 44:1194–201. 10.1086/513201 17407038

[3] Foo NP Chen KT Lin HJ Guo HR. Characteristics of pyogenic liver abscess patients with and without diabetes mellitus. *Am J Gastroenterol*. (2010) 105:328–35. 10.1038/ajg.2009.586 19826410

[4] Gu L Wang Y Wang H Xu D. Analysis of clinical and microbiological characteristics of invasive *Klebsiella pneumoniae* liver abscess syndrome. *BMC Infect Dis*. (2025) 25:626. 10.1186/s12879-025-10981-9 40301787 PMC12039297

[5] Siu LK Yeh KM Lin JC Fung CP Chang FY. *Klebsiella pneumoniae* liver abscess: a new invasive syndrome. *Lancet Infect Dis*. (2012) 12:881–7. 10.1016/S1473-3099(12)70205-0 23099082

[6] Yin D Ji C Zhang S Wang J Lu Z Song X Clinical characteristics and management of 1572 patients with pyogenic liver abscess: a 12-year retrospective study. *Liver Int*. (2021) 41:810–8. 10.1111/liv.14760 33314531 PMC8048845

[7] Zhu J Wang G Xi W Shen Z Wei Q Fang X Lactate promotes invasive *Klebsiella pneumoniae* liver abscess syndrome by increasing capsular polysaccharide biosynthesis via the PTS-CRP axis. *Nat Commun*. (2025) 16:6057. 10.1038/s41467-025-61379-9 40593854 PMC12216495

[8] Feng C Di J Jiang S Li X Hua F. Machine learning models for prediction of invasion *Klebsiella pneumoniae* liver abscess syndrome in diabetes mellitus: a singled centered retrospective study. *BMC Infect Dis*. (2023) 23:284. 10.1186/s12879-023-08235-7 37142976 PMC10157913

[9] Wang Q Duan S Deng S Yu S. Isolated retrobulbar optic neuritis after *Klebsiella pneumoniae* infection: a rare case report and literature review. *IDCases*. (2024) 38:e02106. 10.1016/j.idcr.2024.e02106 39524377 PMC11550208

[10] Ishag MY Alsuleimani AL. Invasive *Klebsiella pneumoniae* causing concurrent liver and pulmonary abscesses: successful management with prolonged oral amoxicillin-clavulanate. *Cureus*. (2025) 17:e87412. 10.7759/cureus.87412 40772223 PMC12327917

[11] Zhang L Chen J Qu Y Cao X Cui J Li J Development and validation of a predictive model for invasive syndrome in patients with *Klebsiella pneumoniae* liver abscess. *Front Med*. (2025) 12:1663407. 10.3389/fmed.2025.1663407 41048965 PMC12488649

[12] Feng CY Zhang LW Liu T Jiang SF Li XM Di J. [Establishment and verification of invasion syndrome prediction model in patients with diabetes complicated with *Klebsiella pneumoniae* liver abscess]. *Zhonghua Yi Xue Za Zhi.* (2024) 104:956–62. 10.3760/cma.j.cn112137-20231019-00813 38514345

[13] Gupta A Bhatti S Leytin A Epelbaum O. Novel complication of an emerging disease: invasive *Klebsiella pneumoniae* liver abscess syndrome as a cause of acute respiratory distress syndrome. *Clin Pract*. (2018) 8:1021. 10.4081/cp.2018.1021 29441188 PMC5806497

[14] Li LH Ahmad R Tanone R Sharma AKSTB. synthetic minority oversampling technique for tree-boosting models for imbalanced datasets of intrusion detection systems. *PeerJ Comput Sci*. (2023) 9:e1580. 10.7717/peerj-cs.1580 38077567 PMC10703015

[15] Xu Z Shen D Kou Y Nie TA. Synthetic minority oversampling technique based on gaussian mixture model filtering for imbalanced data classification. *IEEE Trans Neural Netw Learn Syst*. (2024) 35:3740–53. 10.1109/TNNLS.2022.3197156 35984792

[16] Mullah MAS Hanley JA Benedetti A. LASSO type penalized spline regression for binary data. *BMC Med Res Methodol*. (2021) 21:83. 10.1186/s12874-021-01234-9 33894761 PMC8070328

[17] World Medical Association. World Medical Association Declaration of Helsinki: ethical principles for medical research involving human subjects. *JAMA.* (2013) 310:2191–4. 10.1001/jama.2013.281053 24141714

[18] Wang F Yu J Chen W Mo Z Zhang Y. Clinical characteristics of diabetes complicated by bacterial liver abscess and nondiabetes-associated liver abscess. *Dis Markers*. (2022) 2022:7512736. 10.1155/2022/7512736 35521637 PMC9064492

[19] Schmitz T Freuer D Linseisen J Meisinger C. Associations between blood markers of glucose metabolism and characteristics of circulating lymphocytes. *Clin Nutr*. (2024) 43:285–95. 10.1016/j.clnu.2024.11.004 39546924

[20] Berbudi A Rahmadika N Tjahjadi AI Ruslami R. Type 2 diabetes and its impact on the immune system. *Curr Diabetes Rev*. (2020) 16:442–9. 10.2174/1573399815666191024085838 31657690 PMC7475801

[21] Frydrych LM Bian G O’Lone DE Ward PA Delano MJ. Obesity and type 2 diabetes mellitus drive immune dysfunction, infection development, and sepsis mortality. *J Leukoc Biol*. (2018) 104:525–34. 10.1002/JLB.5VMR0118-021RR 30066958

[22] Lin JC Siu LK Fung CP Tsou HH Wang JJ Chen CT Impaired phagocytosis of capsular serotypes K1 or K2 *Klebsiella pneumoniae* in type 2 diabetes mellitus patients with poor glycemic control. *J Clin Endocrinol Metab*. (2006) 91:3084–7. 10.1210/jc.2005-2749 16720670

[23] Lin YT Wang FD Wu PF Fung CP. *Klebsiella pneumoniae* liver abscess in diabetic patients: association of glycemic control with the clinical characteristics. *BMC Infect Dis*. (2013) 13:56. 10.1186/1471-2334-13-56 23363608 PMC3568401

[24] Steinbach D Ahrens PC Schmidt M Federbusch M Heuft L Lübbert C Applying machine learning to blood count data predicts sepsis with ICU admission. *Clin Chem*. (2024) 70:506–15. 10.1093/clinchem/hvae001 38431275

[25] Adelman MW Casarin S Kurian J Miller WR Connor A Hsu E Platelets and mortality in bloodstream infection: a multicenter cohort study. *Clin Microbiol Infect*. (2025) 31:1733–6. 10.1016/j.cmi.2025.07.021 40744277 PMC12377423

[26] Li H Zhou Y Zhang X Yao R Li N. The relationship between hemoglobin, albumin, lymphocyte, and platelet (HALP) score and 28-day mortality in patients with sepsis: a retrospective analysis of the MIMIC-IV database. *BMC Infect Dis*. (2025) 25:333. 10.1186/s12879-025-10739-3 40065235 PMC11892195

[27] Song Z Li H Zhang J Huang Y Gao S. Platelet traits and sepsis risk and prognosis: a bidirectional two-sample mendelian randomization study. *Shock.* (2025) 63:520–6. 10.1097/SHK.0000000000002447 39158958

[28] Zeng T Sun Y Chen S Pang J Wang H Cai X The causal relationship between blood cell indices and 28-day mortality in sepsis: a retrospective study and bidirectional Mendelian randomization analysis. *BMC Infect Dis*. (2024) 24:619. 10.1186/s12879-024-09532-5 38909204 PMC11193192

[29] Leung G Middleton EA. The role of platelets and megakaryocytes in sepsis and ARDS. *J Physiol*. (2024) 602:6047–63. 10.1113/JP284879 39425883

[30] Yang M Jiang H Ding C Zhang L Ding N Li G STING activation in platelets aggravates septic thrombosis by enhancing platelet activation and granule secretion. *Immunity.* (2023) 56:1013–26.e6. 10.1016/j.immuni.2023.02.015. 36944334

[31] Kubo K Sakuraya M Sugimoto H Takahashi N Kano KI Yoshimura J Benefits and harms of procalcitonin- or C-reactive protein-guided antimicrobial discontinuation in critically Ill adults with sepsis: a systematic review and network meta-analysis. *Crit Care Med*. (2024) 52:e522–34. 10.1097/CCM.0000000000006366 38949476

[32] Hamilton FW Thomas M Arnold D Palmer T Moran E Mentzer AJ Therapeutic potential of IL6R blockade for the treatment of sepsis and sepsis-related death: a Mendelian randomisation study. *PLoS Med*. (2023) 20:e1004174. 10.1371/journal.pmed.1004174 36716318 PMC9925069

[33] Dark P Hossain A McAuley DF Brealey D Carlson G Clayton JC Biomarker-guided antibiotic duration for hospitalized patients with suspected sepsis: the ADAPT-sepsis randomized clinical trial. *JAMA*. (2025) 333:682–93. 10.1001/jama.2024.26458 39652885 PMC11862976

[34] Lei J Zhai J Zhang Y Qi J Sun C. Supervised machine learning models for predicting sepsis-associated liver injury in patients with sepsis: development and validation study based on a multicenter cohort study. *J Med Internet Res*. (2025) 27:e66733. 10.2196/66733 40418571 PMC12149780

[35] Gou E Yang Q Chen J Kong T Tang Z Wen Q Association between albumin-bilirubin score and in-hospital mortality in patients with sepsis: evidence from two large databases. *Heliyon*. (2024) 10:e34697. 10.1016/j.heliyon.2024.e34697 39170393 PMC11336323

[36] Ye D Jiang W Gu D. Association between platelet-albumin-bilirubin grade and the 30-day mortality in patients with acute respiratory distress syndrome: evidence from the MIMIC-IV Database. *Balkan Med J*. (2025) 42:66–74. 10.4274/balkanmedj.galenos.2024.2024-8-7 39757517 PMC11725668

[37] Song H Wang X Lian Y Wan T. Analysis of the clinical characteristics of 202 patients with liver abscess associated with diabetes mellitus and biliary tract disease. *J Int Med Res*. (2020) 48:300060520949404. 10.1177/0300060520949404 32865074 PMC7469731

[38] Shi S Xia W Guo H Kong H Zheng S. Unique characteristics of pyogenic liver abscesses of biliary origin. *Surgery*. (2016) 159:1316–24. 10.1016/j.surg.2015.11.012 26775071

[39] Große K Ohm D Würstle S Brozat JF Schmid RM Trautwein C Clinical characteristics and outcome of patients with enterococcal liver abscess. *Sci Rep*. (2021) 11:22265. 10.1038/s41598-021-01620-9 34782684 PMC8593075

[40] Mücke MM Kessel J Mücke VT Schwarzkopf K Hogardt M Stephan C The role of Enterococcus spp. and multidrug-resistant bacteria causing pyogenic liver abscesses. *BMC Infect Dis*. (2017) 17:450. 10.1186/s12879-017-2543-1 28651522 PMC5485679

[41] Chen WH Chiu CH Huang CH Lin CH Sun JH Huang YY Pyogenic liver abscess caused by *Pseudomonas aeruginosa*: clinical analysis of 20 cases. *Scand J Infect Dis*. (2011) 43:877–82. 10.3109/00365548.2011.599332 21867474

[42] Lee SW Tsai CA Lee BJ. Chryseobacterium meningosepticum sepsis complicated with retroperitoneal hematoma and pleural effusion in a diabetic patient. *J Chin Med Assoc*. (2008). 71:473–6. 10.1016/S1726-4901(08)70151-5 18818141

[43] Petri CR Majid A Anandaiah AA. Man with biliary sepsis and an enlarging pleural effusion. *Ann Am Thorac Soc*. (2019) 16:496–8. 10.1513/AnnalsATS.201809-622CC 30932703

[44] Huang CH Wang SF Lee CH Wu YM Chang C Chen BH Bacteremia (Sepsis), hepatorenal syndrome, and serum creatinine levels rather than types or microbial patterns predicted the short-term survival of cirrhotic patients complicated with spontaneous bacterial peritonitis. *Diagnostics*. (2022) 13:94. 10.3390/diagnostics13010094 36611386 PMC9818281

