# Correction: Development and validation of a prediction model for invasive syndrome in liver abscess patients based on LASSO regression: a multi-center retrospective cohort study in China

**DOI:** 10.3389/fmed.2025.1756791

**Published:** 2026-01-05

**Authors:** Dian-Dian Hao, Guang-Zhao Shao, Xiu-Li Wang, Chang-Cheng Zhao, Jia-Lin Du, Hui Wang, Yu-Lin Ren, Yu-Ze Song, Xiao-Yu Wen

**Affiliations:** 1 Department of Hepatology, Center for Infectious Diseases and Pathogenic Biology, The First Hospital of Jilin University, Changchun, China; 2 Department of Hepatobiliary and Pancreatic Surgery, General Surgery Center, The First Hospital of Jilin University, Changchun, China; 3 Department of General Surgery, Yanshi People's Hospital, Luoyang, Henan, China; 4 Department of General Surgery, The First Affiliated Hospital of Zhengzhou University, Zhengzhou, Henan, China

**Keywords:** liver abscess, invasive syndrome, multicenter study, predictive model, web-based calculator

An incorrect **Funding** statement was provided. The correct funder is: The Foundation of Science and Technology Commission of Jilin Province (Grants No. YDZJ202401181ZYTS) the correct funding statement reads:

The author(s) declared that financial support was received for this work and/or its publication. This study was supported by the Foundation of Science and Technology Commission of Jilin Province (Grant No. YDZJ202401181ZYTS).

The original version of this article has been updated.

