# Supplementary material for: Development and validation of a prediction model for invasive syndrome in liver abscess patients based on LASSO regression: a multi-center retrospective cohort study in China
Source: Front Med (Lausanne). 2025 Nov 28;12:1600509. doi: 10.3389/fmed.2025.1572054 (PMC12698520; doi:10.3389/fmed.2025.1572054)
Supplement: Supplementary file 1 [file Supplementary_file_1.docx]

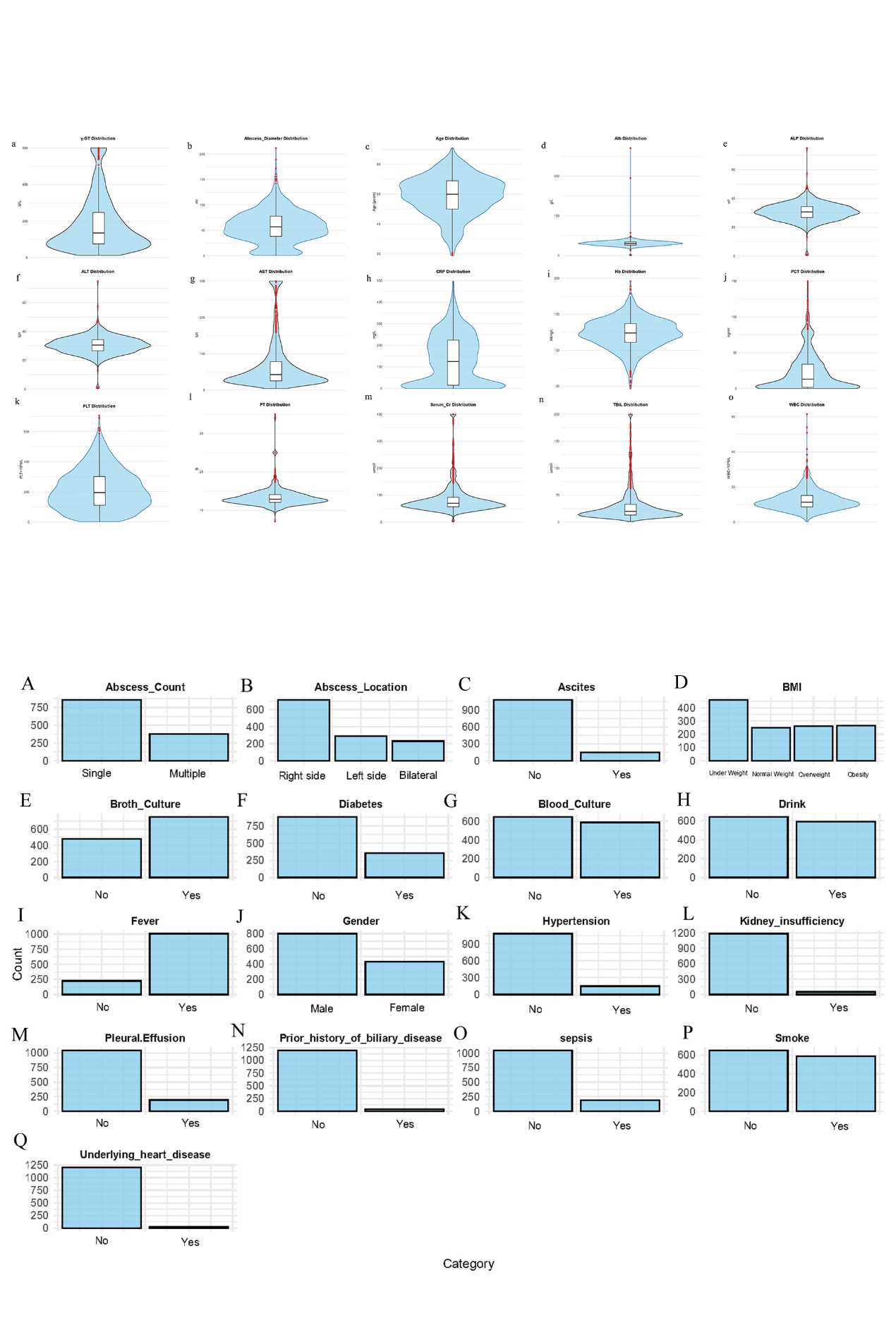


**Supplementary Figure 1: Raw Data Distribution Plot.** Continuous variables: a, γ-GT; b, Abscess Diameter; c, Age; d, Alb; e, ALP; f, ALT; g, AST; h, CRP; i, Hb; j, PCT; k, PLT; l, PT; m, Serum Cr; n, TBIL; o, WBC. Categorical variables: A, Abscess count; B, Abscess location; C, Ascites; D, BMI; E, Broth culture; F, Diabetes; G, Blood culture; H, Drink; I, Fever; J, Gender; K, Hypertension; L, Kidney insufficiency; M, Pleural effusion; N, Prior history of biliary disease; O, Sepsis; P, Smoke; Q, Underlying Heart Disease. γ-GT: Gamma-Glutamyl Transferase; Alb: Albumin; ALP: Alkaline Phosphatase; ALT: Alanine Aminotransferase; AST: Aspartate Aminotransferase; CRP: C-Reactive Protein; Hb: Hemoglobin; PCT: Procalcitonin; PLT: Platelet Count; PT: Prothrombin Time; Serum Cr: Serum Creatinine; TBIL: Total Bilirubin; WBC: White Blood Cell Count; BMI: Body Mass Index.


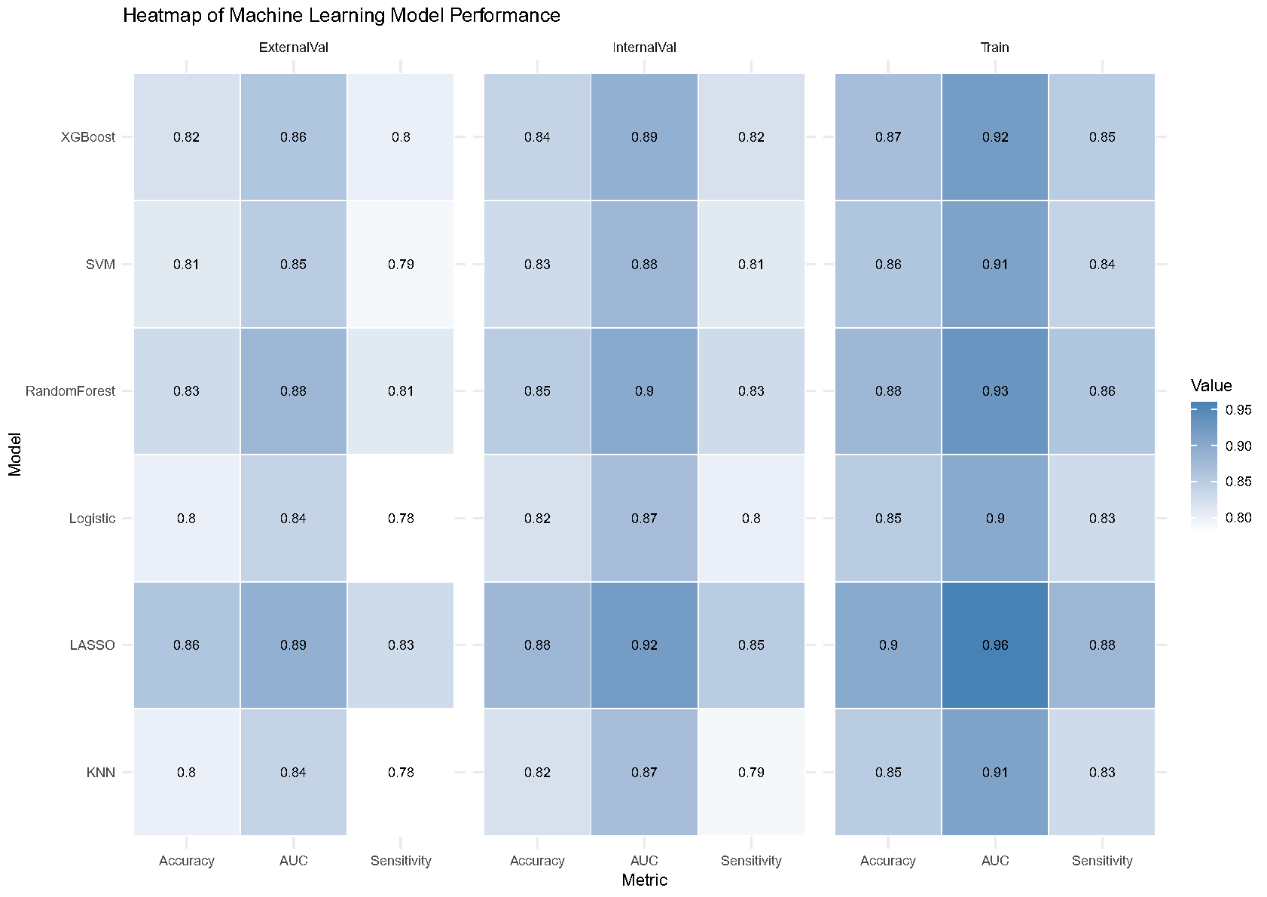


**Supplementary Figure 2:** Comparison of Model Prediction Performance Illustrated by Heatmap.


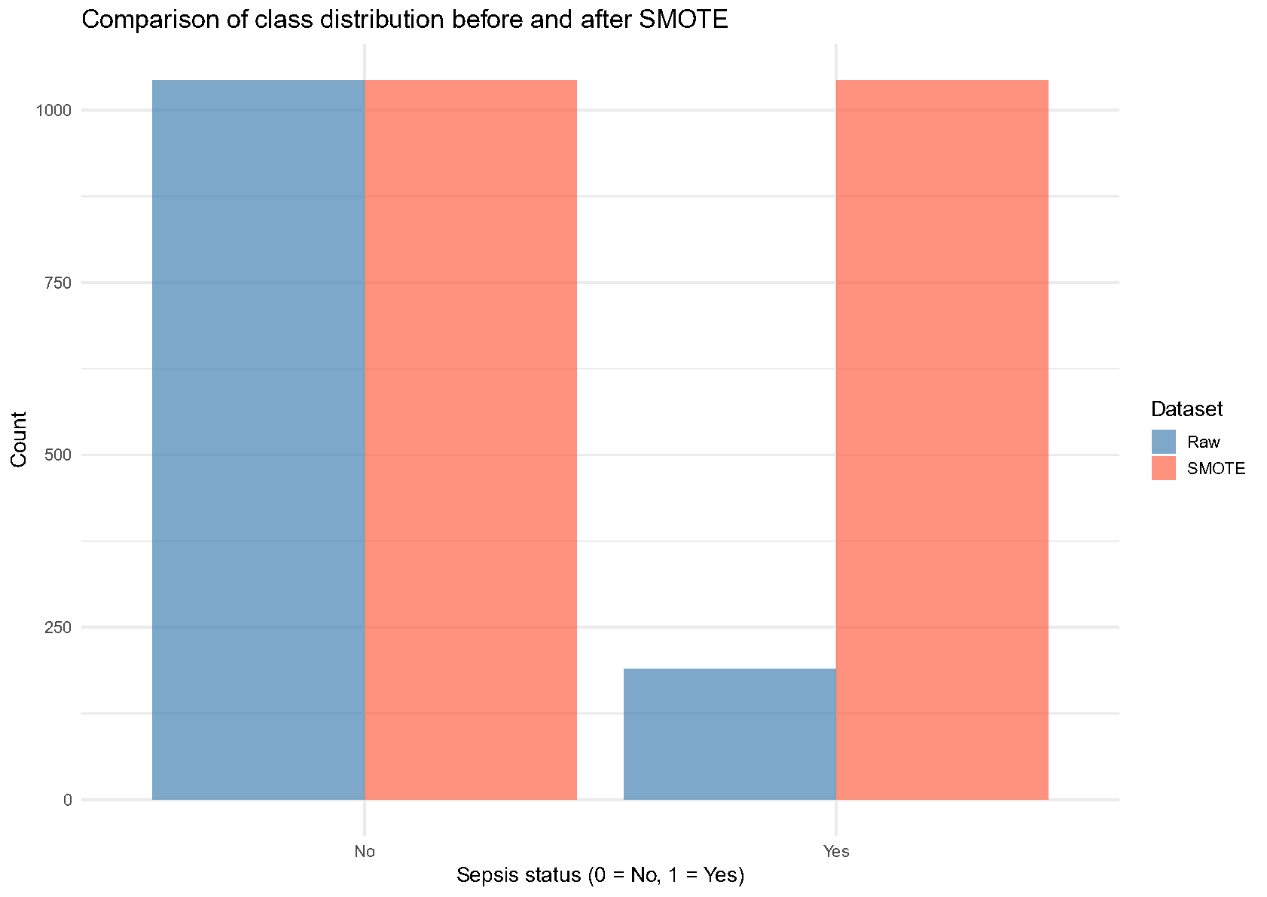


**Supplementary Figure 3:** Distribution of Sepsis Outcome. Distribution of Sepsis (yes/no) in the training datasets before and after SMOTE oversampling.

Web-based Calculator Implementation Code

install.packages("DynNom") # Install the package

library(DynNom) # Load the package

install.packages("survival") # Install the package

library(survival) # Load the package

data(lung) # Load the built-in R dataset 'lung'

# Build a survival prediction model

mod <- coxph(Surv(time, status) ~ age + strata(sex) + ph.ecog, data = lung)

DynNom(mod) # Generate a dynamic nomogram (Dynamic Nomogram)

DNbuilder(mod) # Generate the nomogram file in the working directory

install.packages('rsconnect')

library(rsconnect)
